# Fluorescent and Water Dispersible Single‐Chain Nanoparticles: Core–Shell Structured Compartmentation

**DOI:** 10.1002/anie.202015179

**Published:** 2021-02-25

**Authors:** Justus F. Hoffmann, Andreas H. Roos, Franz‐Josef Schmitt, Dariush Hinderberger, Wolfgang H. Binder

**Affiliations:** ^1^ Macromolecular Chemistry Institute of Chemistry, Faculty of Natural Science II (Chemistry, Physics and Mathematics) Martin Luther University Halle-Wittenberg von-Danckelmann-Platz 4 06120 Halle Germany; ^2^ Physical Chemistry Institute of Chemistry Faculty of Natural Science II (Chemistry, Physics and Mathematics) Martin Luther University Halle-Wittenberg von-Danckelmann-Platz 4 06120 Halle Germany; ^3^ Institute of Physics, Faculty of Natural Science II (Chemistry, Physics and Mathematics) Martin Luther University Halle-Wittenberg von-Danckelmann-Platz 3 06120 Halle Germany

**Keywords:** amphiphiles, decay associated spectra, EPR spectroscopy, fluorescence spectroscopy, nanostructures, single-chain nanoparticles

## Abstract

Single‐chain nanoparticles (SCNPs) are highly versatile structures resembling proteins, able to function as catalysts or biomedical delivery systems. Based on their synthesis by single‐chain collapse into nanoparticular systems, their internal structure is complex, resulting in nanosized domains preformed during the crosslinking process. In this study we present proof of such nanocompartments within SCNPs via a combination of electron paramagnetic resonance (EPR) and fluorescence spectroscopy. A novel strategy to encapsulate labels within these water dispersible SCNPs with hydrodynamic radii of ≈5 nm is presented, based on amphiphilic polymers with additional covalently bound labels, attached via the copper catalyzed azide/alkyne “click” reaction (CuAAC). A detailed profile of the interior of the SCNPs and the labels’ microenvironment was obtained via electron paramagnetic resonance (EPR) experiments, followed by an assessment of their photophysical properties.

## Graphical abstract

The internal structure of single‐chain nanoparticles (SCNPs) has been investigated, forming nanosized domains preformed during the crosslinking process. We present proof for the presence of nano‐compartments within SCNPs via a combination of electron paramagnetic resonance (EPR) and fluorescence spectroscopy. A novel strategy to encapsulate labels within such water dispersible SCNPs is presented.

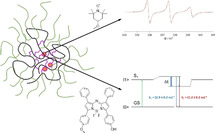

## Introduction

Among many nanosized carriers single chain nanoparticles (SCNPs) are the most versatile, both in molecular design, structural diversity and embedded functionalities.^[1]^ Derived from single polymer chains by covalent or noncovalent crosslinking they directly link the vast synthetic space of controlled polymer synthesis with their function, embedding chemical functionalities into the final SCNPs to allow applications of SCNPs in the areas of catalysis,^[2]^ drug delivery^[3]^ or imaging technologies[^3b^, ^4^] such as photoacoustic imaging.^[5]^ Crucial thereto is the placement of specific chemical functionalities within the SCNP, further stimulated by its nanosized dimension, excellent dispersion and internal compartmentation. The formation of nanosized compartments within such SCNPs has been intensely discussed,[^1a^, ^6^] proposing their use as functional units to generate microenvironments for catalysis,^[7]^ photophysics,^[8]^ or subsequent embedding of chiral elements.^[9]^ As the formation of SCNPs is based on the collapse of single polymer chains at low concentration via covalent or non‐covalent intramolecular bonding interactions,^[10]^ it “freezes‐in” the conformational state of the polymer chain into the final, crosslinked SCNP.^[11]^ However, dynamics of the polymer chains often is at least partially preserved depending on the degree of crosslinking and solvent effects during the crosslinking process.^[6]^ Thus recently a strong solvent dependency on the final size of single‐chain nanoparticles has been observed using comb‐shaped polymers, bearing for example, polyisobutylene (PIB) sidechains with a direct correlation between solvent quality and the final SCNP size.^[8b]^ Strong photophysical effects as for example an increasing rate of photoinduced dimerization has been observed within pre‐folded SCNPs via an increase of quantum yields by the confinement‐effects within the SCNP.^[8b]^ Structural dynamics can lead to a modulation of the band structure of SCNPs and the excited states of bound dye molecules on the sub‐ns timescale which determines the achieved fluorescence quantum yield and both, the absorption and fluorescence spectra of the dyes.^[12]^

Although the formation of nanosized compartments within the SCNPs has been often proposed and observed indirectly, a direct proof has not been accomplished as many of the used methodologies provide an only indirect measure of compartmentation. Thus monitoring the molecular dynamics of water molecules around SCNPs via Overhauser dynamic nuclear polarization (ODNP) hinted to supramolecular organized hydrophobic benzene‐1,3,5‐tricarboxamides (BTA) into chiral folds within the SCNP, resembling their self‐assembly in solution.^[13]^ Also the solution behavior of polymers within SCNPs has been shown to deviate from those of the respective uncrosslinked polymers due to confinement within nanosized compartments, leading to significant changes in their lower critical solution (LCST) behavior.^[14]^

We here probe the formation of compartments within synthetic SCNPs based on amphiphilic polymers, equipped with an active spin label, via continuous wave electron paramagnetic wave resonance (CW EPR), allowing to probe the internal compartments of the formed SCNPs. Additionally, fluorescent dyes, appropriate for use in pump‐probe photoacoustic imaging^[15]^ are embedded into small (*r*_h_<10 nm) SCNPs to allow sufficient permeation for cell‐incorporation.^[16]^ Thus the SCNPs act as a barrier against defense mechanisms of the targeted tissues and stabilize the dye against external photobleaching. Important in our endeavor was the quest to position the dye within the hydrophobic core of the SCNP, as such its accessibility is reduced and thus a proposed protection effect of the SCNP could be expected to be most effective.

With the here reported single‐chain nanoparticles one can therefore generate a scaffold for encoding the required different functionalities stimuli‐response, for example, thermo‐ or pH‐responsivity,[^3c^, ^17^] and biocompatibility,^[18]^ by using three types of monomers to adjust both, the required amphiphilic balance for the single‐chain collapse, and the dye attachment.

## Results and Discussion

**Scheme 1:**
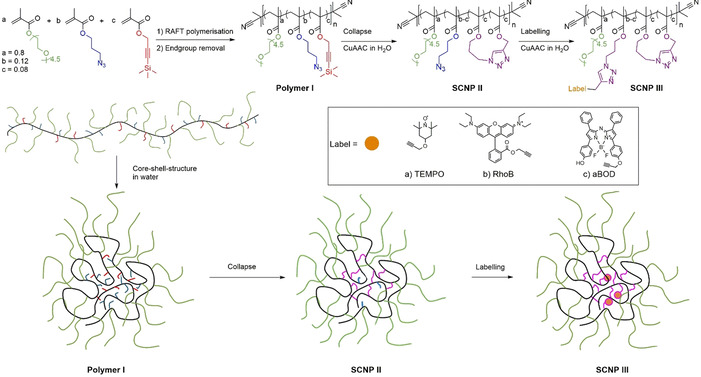
Synthesis of the precursor **polymer I** by RAFT polymerization followed by the formation of the unlabeled **SCNP II** and labelling to obtain the labelled **SCNP IIIa‐c**.

The required poly(poly(ethylene glycol) methacrylate) (PEGMA)‐copolymer (**polymer I**) was synthesized by RAFT copolymerization of poly(ethylene glycol) methacrylate (*M_n_*=300 Da, n=4.5), azidopropyl methacrylate, and 3‐(trimethylsilyl)propargyl methacrylate in DMF, using AIBN as initiator and cyanoisopropyl dithiobenzoate (CPDB) as chain transfer agent (CTA), followed by the removal of the CTA using an excess of AIBN.^[19]^ The CTA,^[20]^ the monomers,^[21]^ and the dye labels^[22]^ were synthesized according to literature with small adaptions (see Supporting Information). For the polymerization the molar fractions of the three monomers were set as 0.8, 0.12, and 0.08, respectively, to achieve an appropriate number of crosslinking groups^[23]^ and to retain residual attachment sites for modification with the labels after the single‐chain collapse (**polymer I**: *M_n_*=36.1 kDa, *PDI*=1.7, *DP*=129). Furthermore, this composition allows to induce thermoresponsivity together with sufficient dispersibility and hydrophobic packaging of the labels. Final composition and structural integrity of the resulting polymer was proven by NMR‐spectroscopy, allowing to adjust the desired functionalities along the polymer chain in relation to the initially used amounts of the three monomers (see Supporting Information, including the final copolymer‐compositions). Subsequently, single‐chain collapse of **polymer I** and labelling (see Scheme 1) was accomplished in a two‐step/one‐pot reaction, where the alkyne group was first deprotected by tetra butyl ammonium fluoride in water, followed by slow addition of the resulting polymer solution to an aqueous CuSO_4_/NaAsc solution via a syringe pump to adjust low concentrations (*c*_polymer_<10^−6^ M) for the required single‐chain folding. The amphiphilic polymer thus is adopting a preorganized nanoparticle by forming an intramolecular core–shell structure with the hydrophilic PEG sidechains in the shell and the reactive azide‐groups in the core, resulting in sole intrachain crosslinking of the reactive groups to form **SCNP II** via CuAAC‐reaction (see Scheme 1).^[24]^ For further labelling of **SCNP II**, the catalyst was reactivated by adding additional sodium ascorbate and a label‐alkyne solution in water (with the help of Kolliphor EL as detergent for the 2,2,6,6‐tetramethylpiperidine oxide (TEMPO)‐label and aza‐BODIPY (aBOD)). This allows a reaction of the still present residual terminal azido moieties in the hydrophobic core to attach the two fluorescent dyes Rhodamine B (RhoB) and aBOD as a NIR‐fluorescent dye. Especially aBOD is attractive as it displays a tumor targeting functionality via deprotonation, resulting in intramolecular charge transfer, and thereby suppression of fluorescence.[^22d^, ^25^] The EPR‐label 2,2,6,6‐tetramethylpiperidine oxide (TEMPO) was attached for subsequent EPR investigations of the SCNP's inner core (see Scheme 1).

Progress of the crosslinking was followed by attenuated total reflection infrared (ATR‐IR) spectroscopy (see Figure S1a and b) indicating a full disappearance of the alkyne band at 2178 cm^−1^ in the **SCNP II**, and a partial and complete removal of the azide band at 2100 cm^−1^ for **SCNP II** and the **SCNPs III**, respectively. The new bands of the triazole ring are overlapped by the bands of the polymer backbone and the PEG sidechains and therefore not visible in the spectrum. ^1^H‐NMR spectroscopy proves a complete reaction of the reactive groups under formation of triazole rings (see Figure S1d, removal of the TMS‐protecting group at 0.2 ppm, absence of the alkyne proton at 2.6 ppm). A crosslinking density of ≈9 crosslinks per SCNP was roughly calculated from the stoichiometric ratios, leaving ≈4 remaining attachment sites per SCNP for the labels. The so obtained SCNPs are water dispersible and can be redispersed after freeze‐drying.

**Figure 1:**
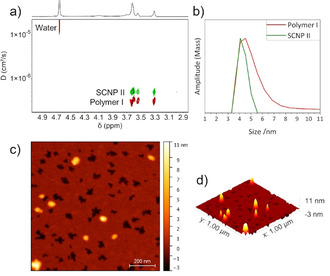
a) DOSY‐NMR and b) DLS measurements of the precursor **polymer I** and the unlabelled **SCNP II** at concentrations of 1 mg mL^−1^ in water and D_2_O, respectively. c) 2D and d) 3D AFM topography image of **SCNP IIIb** (1×1 μm).

Diffusion‐ordered NMR spectroscopy (DOSY‐NMR) and dynamic light scattering measurements of **polymer I** and **SCNP II** (see Figure 1 and Table 1) independently show a reduction of the hydrodynamic radius after the CuAAC from 5.2 nm to 4.2 nm. This proves the single‐chain collapse in good agreement with literature, where a reduction of the hydrodynamic radius is an indication of a single‐chain collapse.^[26]^ Upon further labelling of the **SCNP II** yielding **SCNPs IIIa‐c**, both methods show the trend of an increased hydrodynamic radius of ≈1.4 nm (DOSY‐NMR, see Table 1) and of ≈0.5 nm (DLS) indicating the successful attachment of the respective labels, which lead to an expansion of the measured volume, as the dye‐labels are comparably large in dimension. Atomic force microscopy (AFM) further proved the formation of SCNPs with an average height of 7.5±1.5 nm (see Figure 1 c,d and Figure S2). This underscores the formation of distorted particles due to the surface/SCNP‐interaction between the polar mica‐surface and the hydrophilic PEG‐shell, also indicative of the high responsivities of the SCNPs during adsorption, inline with previous observations.^[27]^

**Table 1:** The hydrodynamic radii (*r*_h_) and cloud point temperatures (*T*_cp_) of the precursor **polymer I**, the unlabeled **SCNP II** and labelled **SCNPs IIIa‐c**, bearing the different labels TEMPO (**IIIa**), RhoB (**IIIb**) and aBOD (**IIIc**).

	*D*_DOSY_^[a]^ [×10^−7^ cm^2^ s^−1^]	*r*_h,DOSY_^[b]^ [nm]	*r*_h,DLS_^[c]^ [nm]	*T*_cp_^[d]^ [°C]
Polymer I	3.3	5.3	5.2±1.9	48
SCNP II	4.9	3.6	4.2±0.4	54
SCNP IIIa (TEMPO)	3.4	5.2	4.9±1.0	65
SCNP IIIb (RhoB)	3.4	5.2	4.5±0.6	69
SCNP IIIc (aBOD)	3.7	4.7	4.6±0.4	75

[a] Diffusion coefficient measured by DOSY‐NMR in D_2_O. [b] Calculated using the Stokes–Einstein equation (*r*_h_=(*k*_B_ *T)*/(6*πηD*) with *D=D*_DOSY_, *η* ${}_{D{}_{2}O}$ =1.25 MPa s). [c] Measured by DLS in H_2_O. [d] Cloud point temperature measured by turbidimetry, *T*_cp_ *=T* at 96 % transmittance (see Figure S3 b).

Size exclusion chromatography (SEC) measurements in water further prove the covalent attachment of the labels to the SCNPs via an overlap of the R.I. and UV/Vis track for the dye‐labelled **SCNPs IIIb** and **IIIc** (see Figure S4 d and e). During single‐chain collapse, the hydrophobic reactive groups (azide/alkyne) are proposed to be located in the inner core of the nanoparticles, hidden from the aqueous surroundings. We therefore observe a polarity shift in SEC, inline with an increased hydrophobic collapse (*T*_cp_) after the single‐chain collapse and with further modification moving from the native **polymer I** (*T*_cp_=48 °C) to **SCNP II** (*T*_cp_=54 °C) to **SCNP IIIa‐c** (*T*_cp_=65 °C, 69 °C and 75 °C, respectively) (see Table 1 and Figure S3). It should be noted that this is the first observation of such a consequent increase of *T_cp_*, initially only observed for noncrosslinked, dynamic random copolymers.^[14]^ Additionally, this behavior explains the decrease of retention time in SEC moving from **polymer I** to **SCNP II**, with concomitantly increasing *T*_cp_. Labelling **SCNP II** with the dyes to yield **SCNPs IIIa‐c** leads to a further decrease of the retention time and a further increase of *T*_cp_.

To achieve a deeper understanding of the microstructure and the distribution and location of the individual polymer and label segments within the SCNPs, CW EPR measurements were conducted. For the protective effect in SCNPs to be effective, the exact location (spatially and in chemical environments) of the labels within the SNCPs and the desired location within the hydrophobic core is crucial. Moreover, a rough quantification of the number of labels in one SCNP is needed. With the CW EPR spectra of the attached TEMPO label it is possible to specify the chemical environment of the nanostructure surrounding the probe within a radius of ≈1 nm.^[28]^ For comparison of the TEMPO‐labelled **SCNP IIIa** with an equally labelled linear polymer, the TEMPO‐labelled **polymer I′a** was synthesized analogously to **polymer I**, but without the alkyne moieties (see Supporting Information).

**Figure 2:**
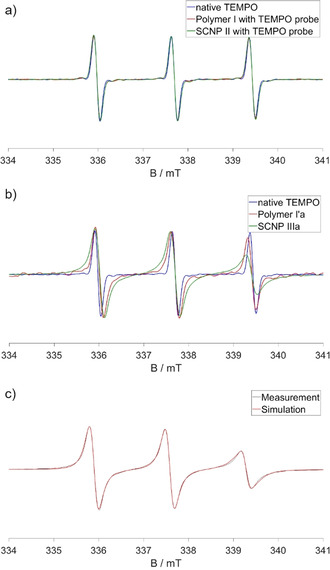
CW EPR spectra of a) native TEMPO, **polymer I** with a TEMPO probe, and **SCNP II** with a TEMPO probe; b) native TEMPO, **polymer I′a**, and **SCNP IIIa**; c) simulated spectrum of **SCNP IIIa**. The spectra were all normalized to the center peak. All measurements were conducted in water at 20 °C. The concentrations of the polymers and SCNPs were 1 mg mL^−1^. The concentrations of the TEMPO probes were 100 μM.

Upon covalently binding TEMPO as a label (Figure 2 b) to **polymer I′** yielding **polymer I′a**, a significant broadening of the EPR peaks is observed, caused by the hindered rotation of the label attached to the large polymer. This effect is even stronger in the TEMPO‐labelled **SCNP IIIa**, indicating a reduction of the mobility of the label upon attachment to the SCNP. Figure 2 a in comparison shows the CW EPR spectra of **polymer I** and **SCNP II** in water in pure physical mixtures with the free TEMPO probe. The three identical normalized spectra indicate free TEMPO in water, proving the absence of specific, mostly hydrophobic interactions of the amphiphilic TEMPO probe with the polymer and the SCNP, respectively, as we usually and regularly observe it with amphiphilic (LCST‐type) polymers.^[28a]^ Simulated spectra of the samples quantify the mobility reduction and the low polarity of the environment as expected for water‐depleted, polymer‐rich regions (see Figure 2 c). For **SCNP IIIa**, but not for the respective polymers an exchange coupling of 6 MHz had to be included to the calculation, indicating high local concentrations and direct contact of two or more TEMPO labels with distances <1 nm. Even though a certain amount of TEMPO was probably deactivated by reduction during the labelling process because of sodium ascorbate present in the reaction mixture, the degree of labelling was approximated to be 1.3 active labels per particle (see Figure S10). This, together with our spectra, leads to the conclusion that the SCNPs bear one to two chemically attached active spin labels and even more EPR‐inactive labels.

**Figure 3:**
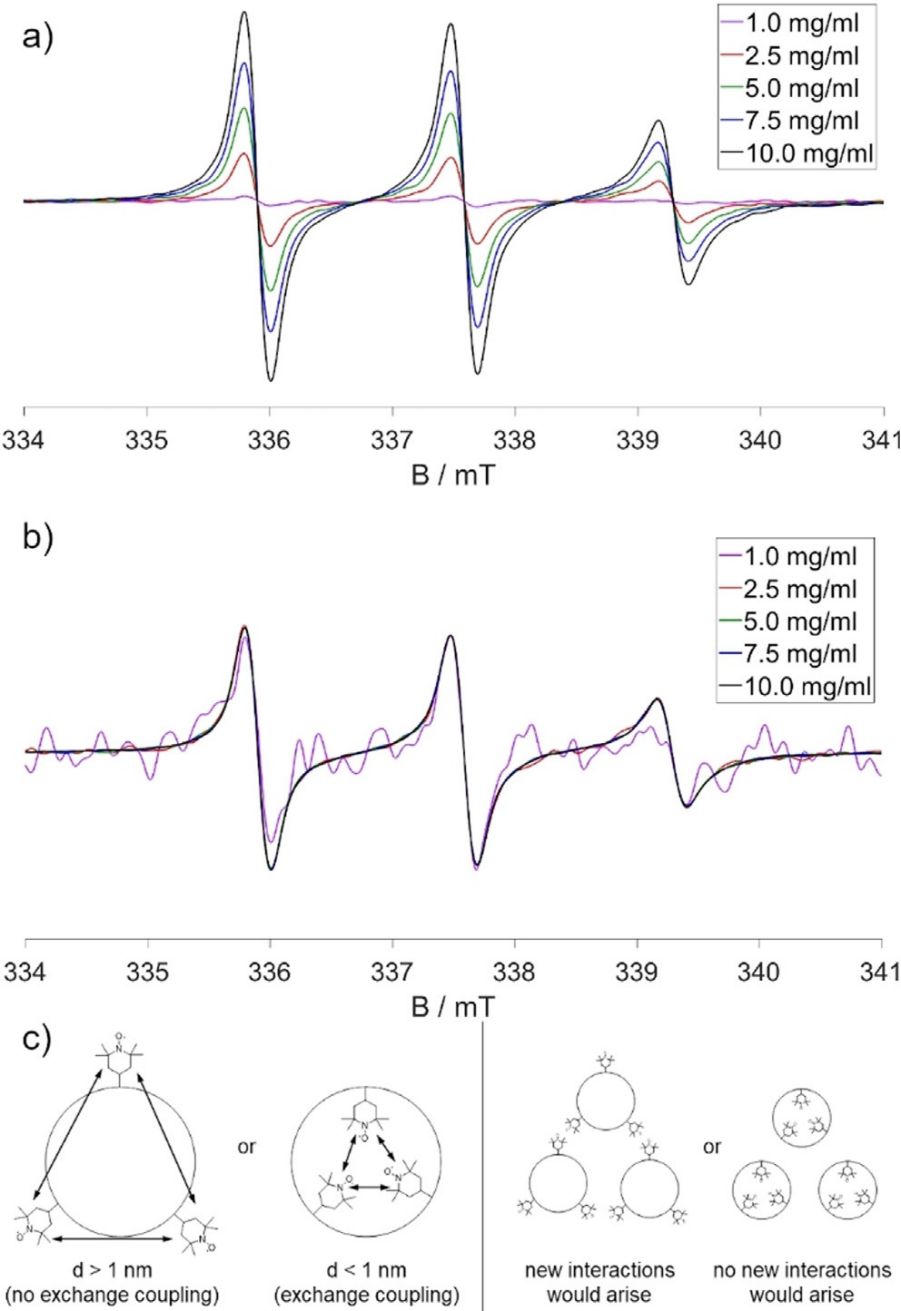
CW EPR spectra of **SCNP IIIa** at different concentrations: a) unnormalized, b) normalized to the center peak. All measurements were conducted in water at 20 °C. c) Schematic depiction of the CW EPR results from the simulation (left side) and the concentration dependence (right side).

Concentration dependent measurements of **SCNP IIIa** in Figure 3 a,b indicate that the label is hidden from the environment outside the nanoparticle as expected for the specific topology and compartmentation in the SCNPs. All normalized spectra show the same shape, apart from small differences in the signal‐to‐noise ratio. Even at high concentrations the surroundings of the labels remain the same. Thus, the label seems to be completely unaffected by external influences outside of the particle, even though collision or agglomerates with other particles at high concentrations might occur (see Figure 3 c). Summing up the EPR results, the up to two active labels per nanoparticle are covalently bound in **SCNP IIIa** in a confined space surrounded by the non‐polar polymer backbone, which is reflected in the lower hyperfine splitting of labels on SCNPs, in the nanoparticles core, altogether as a strong proof for the formation of such a compartment. External influences do not affect the label, indicating its embedding in the SCNP's core. Considering that TEMPO is more hydrophilic than the hydrophobic dyes embedded for optical applications, the SCNPs are therefore able to protect a photoactive label from photooxidation or other degradational processes.

**Figure 4:**
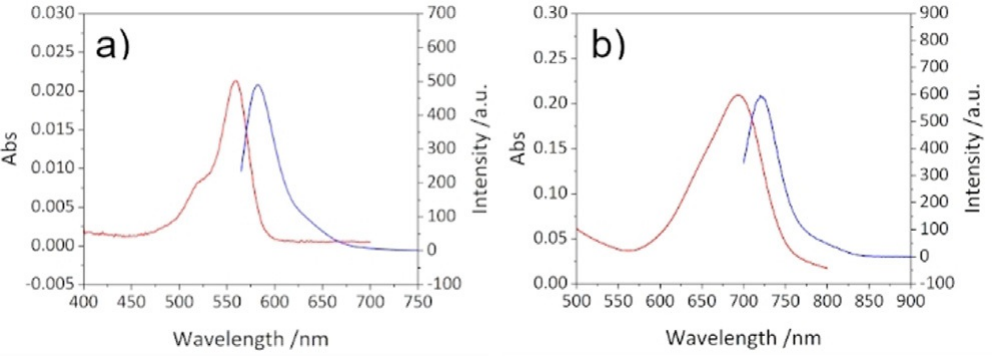
Absorption and fluorescence spectra of a) RhoB‐labelled **SCNP IIIb** in water (*c*=0.5 mg mL^−1^, *λ*_Ex_=559 nm, slit=5 nm, *V*_detector_=600 V), and b) aBOD‐labelled **SCNP IIIc** in aqueous phosphate buffer (pH 6.0), *c*=0.5 mg mL^−1^, *λ*_Ex_=694 nm, slit=5 nm, *V*_detector_=600 V).

The optical properties of dyes can be constructively tuned by binding to SCNPs.[^12^, ^29^] Figure 4 and Table 2 show the absorption and fluorescence spectra and wavelengths of the RhoB and aBOD labelled SCNPs in water and aqueous phosphate buffer (pH 6.0), respectively, spectra of the free dyes can be seen in Figure S5. In comparison to free RhoB‐alkyne, the RhoB‐labelled **SCNP IIIb** shows a red shifted absorption at 559 nm and a blue shifted fluorescence at 582 nm (stokes shift reduced to 23 nm). In the aBOD‐labelled **SCNP IIIc**, resonances are blue shifted by 6 nm in comparison to the free aBOD dye in aqueous phosphate buffer, with a stokes shift of 27 nm for the free aBOD dye and **SCNP IIIc**, respectively. In line with the results from EPR measurements, those shifts occur by a different solvation of the dyes in the particle's hydrophobic core, well separated from the surrounding bulk water phase, again indicative for the compartmented nanostructure of the SCNP.

**Table 2:** Absorption and fluorescence maxima of the fluorescent dyes and the dye‐labelled SCNPs in water.

	RhoB‐alkyne	**SCNP IIIb**	aBOD	**SCNP IIIc**
*λ*_Abs_ [nm]	558	559	700	694
*λ*_Fl_ [nm]	596	582	727	721
Stokes shift [nm]	38	23	27	27
p*K*_a_	–	–	6.8	7.6

The near neighborhood of two or more bound dye molecules leads to interactions and, dependent on their distance and orientation, possible excitonic coupling between the dyes which changes the fluorescence quantum yield, the wavelength maximum and FWHM of the fluorescence emission.^[12]^ In addition internal relaxation processes on the sub‐ns time scale and the interaction of the dye with the polymer at different sites and possible distributions of the molecular structure modulate the fluorescence emission and determine the fluorescence lifetime of excited states.^[30]^ Time resolved fluorescence spectroscopy enables the analysis of sub‐band structures, dynamics of interaction between excited states and the environment and possible heterogeneous decay channels resulting from excitonic coupling and/or compartmentation that contribute to the integral fluorescence spectrum. Just like in the EPR measurements we synthesized a linear aBOD‐labelled polymer (**polymer I′c** see Supporting Information) to compare it to **SCNP IIIc** in view of their excitational behavior and heterogeneity.

**Figure 5:**
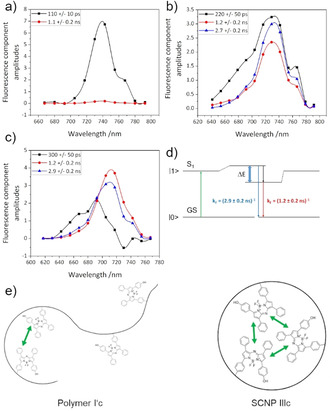
Decay associated spectra measured on a) aBOD in water (*c*=25 μM), b) **polymer I′c** in water (*c*=0.1 mg mL^−1^, dye concentration ca. 5–10 μM) and c) **SCNP IIIc** in water (*c*=0.1 mg mL^−1^, dye concentration ca. 5–10 μM) after Fit with 3 exponential functions (excitation wavelength 632 nm, see SI for further information). d) Excitonic interaction in **SCNP IIIc** and resulting split of the S_1_ states and qualitative band structure due to excitonic splitting and or interaction with the environment of the first excited singlet state (S_1_). The green arrow indicates the excitation from the ground state (GS) and the blue arrow fluorescence from the higher excitonic level with a time constant of 2.9 ns while fluorescence from the lower level decays with 1.2 ns. e) Schematic interpretation of the time resolved fluorescence spectroscopic data of **polymer I′c** (left side) and **SCNP IIIc** (right side).

Figure 5 reveals how an excited state heterogeneity that might either result from molecular coupling and/or a structural heterogeneity, possibly with subsequent relaxation dynamics after light absorption in the bound dyes, leads to a split of the formerly homogeneous excited singlet states of different molecules as indicated in Figure 5 d. While the free aBOD quickly decays with a time constant of 110 ps in water due to quenching of the surrounding aqueous medium the fluorescence lifetime and fluorescence quantum yield significantly rises after binding the dye to a polymer or SCNP. For **polymer I′c** one can see a strong heterogeneity in the decay associated spectra (DAS, Figure 5 b) with a spectral separation of two states with distinct different lifetimes of 1.2±0.2 ns (red curve, energetically lower level) and 2.7±0.2 ns (blue curve, energetically higher level) additionally to a fast decay component that is similar to the free dye (black curve, 220±50 ps) with contributions from molecular interaction (see below). This is caused by a weak and dynamic compartmentation, as depicted in Figure 5 e, with partially folded regions in which the dye molecules can have direct contact to each other and free unfolded regions without direct contact between dye molecules that can be quenched by the aqueous surrounding. This heterogeneity between the bound dye molecules also explains the strong inhomogeneous broadening of the absorption band in Figure S6. The absorption is therefore even broader as compared to **SCNP IIIc** because the elongated polymer has a larger degree of freedoms as compared to the SCNP. As the fluorescence maxima of all components in the DAS are similar the observed heterogeneity refers to structural differences rather than excitonic interaction of the different dye molecules.

For **SCNP IIIc** a stronger compartmentation can be observed from the DAS (Figure 5 c). The decay components redistribute with distinct different maxima, indicating that absorbed light energy potentially is transferred between strongly coupled dye molecules. Slight spectral separation of the two states which were also observed in **polymer I′c** can be seen. The 1.2±0.2 ns component (red curve) seems to be emitted from the energetically lower level while the 2.9±0.2 ns component (blue curve) results from the energetically higher level. The fastest component slows down further as compared to **polymer I′c** and was measured 300±50 ps with strong spectral asymmetry and negative value at 730 nm which is typical for an excitonic relaxation from strongly blue shifted states with an emission around 700 nm to the observed fluorescence maximum around 720–730 nm.

The possible band structure in the material is described schematically in Figure 5 d. The decay associated spectra in Figure 5 c indicate that excitation from the ground state (GS) is followed by radiative decay from the higher state with a time constant of 2.9 ns while fluorescence from the lower state decays with 1.2 ns. Light absorption and relaxation of the exciton induce a complex dynamic into the surrounding polymer and SCNP environment that might cause an apparent relaxation time of about 300 ps possibly accompanied by rearrangements of different compartments or molecular dynamics/ conformation changes of the polymer.^[30]^

The spectroscopic properties are strongly determined by the exact localization of the states at the binding sites of the aBOD molecules in **SCNP IIIc**. Specific configuration for the bound molecules by specific molecular design of the binding sites in the SCNP would allow for the creation of highly individual optical properties of the coupled aBOD dyes and allow for the fine tuning of color and fluorescence yield lifetime of the bound molecules.

The location of the aBOD dye in the SCNP's core also effects its *pK*_a_ and thereby its pH‐responding functionality. As depicted in Table 2 and Figure S7 the fluorescence of the label is strongly changed by the pH value of the solvent, with a *pK*_a_ of 6.8 for the free aBOD and 7.6 for **SCNP IIIc**. Changing the pH of the **SCNP IIIc** dispersion from 6.0 to 8.6 strongly decreases its fluorescence, by 92 %. The pH‐responsivity has no strong influence on the fluorescence lifetime (see Figure S8 and S9). The lifetimes in both, the free aBOD dye and **SCNP IIIc**, stay nearly unchanged over the whole pH range, prospective for a proper tumor tissue targeting. These data are in compliance with the overall fluorescence yield shown in Figure S7 with stronger amplitude reduction observed for **SCNP IIIc** (see Figure S9, Figure S7d) as compared to aBOD in solution (Figure S8, Figure S7b).

## Conclusion

In summary we could observe the formation of internal compartments within single chain nanoparticles (SCNP's) via EPR‐ and fluorescence spectroscopic methods. The SCNPs are synthesized by using a clickable PEGMA based copolymer synthesized by RAFT polymerization, followed by an intramolecular CuAAC within its hydrophobic core. DLS and DOSY‐NMR measurements proved single‐chain collapse with hydrodynamic radii of 4–5 nm. The water dispersible SCNPs all displayed a *T*_cp_ higher than that of the free chain of the native **polymer I**, indicative for the formation of core–shell structured nanoparticles with the hydrophilic PEG chains in the shell and the hydrophobic groups with the embedded labels located inside the core. The inherent dynamics of the SCNPs allows for a medium‐responsive nanostructure as indicated by their thermoresponsivities, and the formation of contacts between two or more labels in one SCNP. Definite proof of this compartmented model consisting of a core–shell structure was accomplished via EPR measurements, proving the presence of at least two covalently attached labels within a confined space with distances below 1 nm, surrounded by the non‐polar polymer backbone and shielded from outer influences by the particle's shell. Based on the quite unique combination of thermal, EPR and photophysical measurements we could prove the nanocompartmented structure of such SCNPs, consisting of the hydrophobic labels embedded within the corresponding hydrophobic compartments, with the short PEG‐chains generating the outer shell. Coupling between dye molecules and the local environment strongly influences their optical properties giving rise to a strategy that allows for specific molecular design of the optical properties. Due to the so enabled shielding of the fluorescent dyes inside the SCNPs, the fluorescent **SCNPs IIIb** and **IIIc** prospect their application as contrasting agents in photoacoustic imaging, still maintaining their targeting properties uninfluenced by their encapsulation in the SCNPs.

## Conflict of interest

The authors declare no conflict of interest.

## Supporting information

As a service to our authors and readers, this journal provides supporting information supplied by the authors. Such materials are peer reviewed and may be re‐organized for online delivery, but are not copy‐edited or typeset. Technical support issues arising from supporting information (other than missing files) should be addressed to the authors.

Supplementary
