# Supplementary material for: Fluorescent and Water Dispersible Single‐Chain Nanoparticles: Core–Shell Structured Compartmentation
Source: Angew Chem Int Ed Engl. 2021 Feb 25;60(14):7820–7. doi: 10.1002/anie.202015179 (PMC8048794; doi:10.1002/anie.202015179)
Supplement: Supplementary file 1 — Supplementary [file ANIE-60-7820-s001.pdf]

Angewandte Chemie
Eine Zeitschrift der Gesellschaft Deutscher Chemiker

Supporting Information

Fluorescent and Water Dispersible Single-Chain Nanoparticles: Core–Shell Structured Compartmentation

*Justus F. Hoffmann, Andreas H. Roos, Franz-Josef Schmitt, Dariush Hinderberger, and Wolfgang H. Binder**

anie_202015179_sm_miscellaneous_information.pdf

Table of Contents

Experimental Procedures 3
Chemicals 3
Instrumentation and analysis 3
Synthesis and sample preparation 5
Supporting Figures 11
References 18

Experimental Procedures

Chemicals

All chemicals were purchased from Sigma Aldrich except triethylamine (TCI), 3-chloro-1-propanol (TCI), boron trifluoride diethyl etherate (TCI), *p*-hydroxychalcone (TCI), diazabicyclo[5.4.0]undec-7-ene (Fluka), diethylamine (Fluka), propargyltosylate (Fluka), trimethylsilyl propargyl alcohol (abcr), KH_2PO_4 (Roanal), K_2HPO_4 (Th. Geyer), nitromethane (Alfa Aesar) and $CuSO_4$ x 5 H_2O (VEB Laborchemikalien). Before use, azobisisobutyronitrile (AIBN) was freshly recrystalized from methanol and poly(ethylene glycol) methyl ether methacrylate (M_n=300) was passed through a basic AlO_x-coloumn to remove the stabilizer.

Instrumentation and analysis

NMR spectra were measured on an Agilent Technologies 400 MHz VNMRS and 500 MHz DD2 at 27°C. Chemical shifts (δ) are reported in ppm and referred to the solvent residual signal (CDCl3 7.26 ppm for ^{1}H and 77.0 ppm for ^{13}C, methanol-d4 3.31 ppm for ^{1}H and 49.0 ppm for ^{13}C, D_2O 4.66 ppm for ^{1}H).

DOSY measurements were done on an Agilent VNMR DD2 500 MHz (sfrq = 499.727 MHz). The experiment was performed under OpenVnmrJ 1.1 and equipped with a 5 mm PFG One NMR probe, z-gradient and temperature unit (27 °C). Diffusion ordered NMR data were acquired by means of the Agilent pulse program DgcsteSL_cc using a stimulated echo with self-compensating gradient schemes and conventional compensation. The length of the gradient pulse was set to 3.0 ms for ^{1}H in combination with a diffusion period of 300 ms (D_2O). Data were systematically accumulated by linearly varying the diffusion encoding gradients over a range from 2% to 95% for 64 gradient increment values.

DLS measurements were performed on a DLS 802 by Viscotek in 1.5 mL semi-micro cuvettes (PMMA). The laser wavelength was 825 – 832 nm so no interaction between the laser and the dyes can be expected.

AFM measurements were performed on a Multimode AFM (Veeco Instruments Inc., Plainview, NY, USA) equipped with Tapping Mode cantilevers Tap150Al-G (BudgetSensors, Sofia, Bulgaria) with a force constant of 5 N m^{-1}, a nominal resonance frequency of 150 kHz, and a radius of <10 nm. The software for the measurement was NanoScope (Veeco Instruments Inc., Plainview, NY, USA) and Gwyddion 2.55 (freeware, http://gwyddion.net/) for interpretation.

ATR-IR spectra were measured on a Bruker Tensor Vertex 70 equipped with a Golden Gate Heated Diamond ATR Top-plate.

THF-based SEC measurements were performed at 30 °C on a Viscotek GPCmax VE 2001 from Viscotek™ applying a CLM3008 precolumn and a CLM3008 main column. As solvent THF was used and the sample concentration was adjusted to 3 mg·mL^{-1} while applying a flow rate of 1 mL·min^{-1}. For determination of the molecular weights the refractive index of the investigated sample was detected with a VE 3580 RI detector of Viscotek™. External calibration was done using poly(styrene) (PS) standards (purchased form PSS) with a molecular weight range from 1050 to 115000 g mol^{-1}.

Water-based SEC measurements were performed at 25 °C on a Viscotek GPCmax VE 2001 from Viscotek™ applying a PSS SUPREMA 5 µm precolumn and a PSS SUPREMA analytical Linear M 5 µm main column. As solvent water (0.05 % NaN_3, 0.1 M NaCl) was used while applying a flow rate of 1 mL·min^{-1}. The sample was detected with a VE 3580 RI detector of Viscotek™ and a UV Detector Knauer Azura UVD 2.19 .

EPR measurements were done with the Miniscope MS 5000 and the MS 5000 temperature controller (magnettech GmbH, Berlin, Germany). By using the Freiberg Instruments software the spectra are measured with a sweep width of 10 mT, a modulation amplitude of 0.02 mT, a digital RC filter with a time constant of 0.2 s and a microwave attenuation of 20 dB. Each spectrum represents an accumulation of 3 scans. The samples were prepared in Rotilabo® sample vials and convicted for measuring in Blaubrands Micropipettes. For the TEMPO probe experiments TEMPO concentrations of 100 µM were used. The spectra were analysed with the EasySpin package version 5.2.27 for MatLab (Mathworks, Inc.)

UV/VIS/NIR-absorption measurements were performed on a Perkin Elmer LAMBDA 365 UV/Vis Spectrophotometer using Helma analytics quartz glass cuvettes (d = 10 mm).

Fluorescence spectra were measured on a Cary Eclipse fluorescence spectrometer of Agilent using Helma analytics quartz glass cuvettes (d = 10 mm).

Turbidimetry measurements were performed using a MP80 Melting Point System by Mettler Toledo. The heating rate was 1 K/min. The polymer concentration was 1 mg/mL.

Decay associated spectra were recorded employing a Hamamatsu R5900 16-channel multi-anode photomultiplier tube (PMT) with 16 separate output (anode) elements and a common cathode and dynode system (PML-16C, Becker&Hickl, Berlin, Germany) as described in Schmitt et al. 2020. A 632 nm pulsed laser diode (PDL-600, Becker&Hickl, Berlin) delivering 80 ps FWHM pulses at a repetition rate of 20 MHz was used for excitation. The fluorescence was observed via a 633 nm longpass filter (F76-631, AHF Analysentechnik, Tübingen, Germany). The determination of the DAS is described in detail in Schmitt et al. 2019.

Synthesis and sample preparation

Synthesis of TEMPO-alkyne

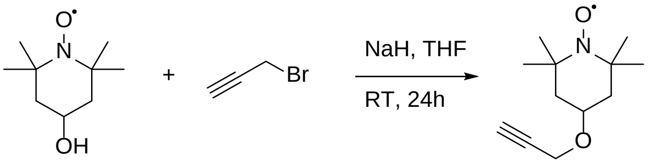

Sodium hydride (60% suspension in mineral oil, 10 mmol, 400 mg) was placed in a Schlenk flask under nitrogen atmosphere and 20 mL of dry THF were added. A solution of *p*-hydroxy-TEMPO (8.71 mmol, 1.5 g) in 5 mL of dry THF was added slowly to the stirring suspension at 0°C. After stirring for 30 min at 0°C propargyl bromide (80% solution in toluene, 11 mmol, 1.0 mL) was added dropwise. The solution was stirred at room temperature for one day and then poured into 150 mL of ice water. The product was extracted with ethyl acetate. The combined organic phases were again washed with water and brine, dried over Na_2SO_4, filtered and evaporated under vacuum. The residue was purified by column chromatography (ethyl acetate: hexane 1:2, R_f = 0.45) to get the product as an orange powder. Yield: 71%. ^{1}H-NMR ($CDCl_3$, 400 MHz, δ in ppm): 4.11 (s, 2H, OCH_2), 2.46 (s, 1H, ≡C*H*) (due to paramagnetic broadening of the nitroxide radical not all signals were observable).

Synthesis of Rhodamine B propargyl ester (RhoB-alkyne)

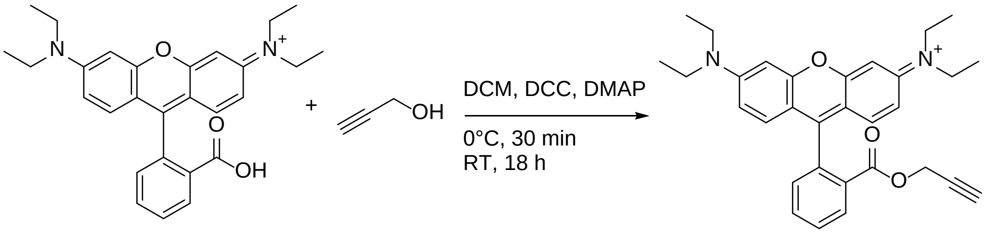

Rhodamine B (1 mmol, 146 mg), dicyclohexylcarbodiimid (1.1 mmol, 226.96 mg) and propargyl alcohol (1.1 mmol, 61.67 mg, 63.5 µL) were dissolved in dry DCM (5 mL) in a Schlenk tube under nitrogen atmosphere. The tube was wrapped in aluminium foil and put into an ice bath. 4-(Dimethylamino)-pyridine (0.2 mmol, 24.43 mg) was added. The reaction mixture was stirred at 0°C for 30 min and at room temperature over night. The colourless precipitate was removed by filtration and washed with DCM. The DCM was removed in vacuum. The crude product was dissolved in ACN, filtered and dried under vacuum. The product was purified by column chromatography (methanol:chloroform 1:5, R_f = 0.4) to get the product as a golden powder. Yield: 40%. ^{1}H-NMR ($CDCl_3$, 500 MHz, δ in ppm): 8.31 (1H, m, H_{Ar}), 7.84 (1H, m, H_{Ar}), 7.75 (1H, m, H_{Ar}), 7.35 (1H, m, H_{Ar}), 7.06 (2H, m, H_{Ar}), 6.91 (2H, m, H_{Ar}), 6.86 (2H, m, H_{Ar}), 4.61 (2H, d, J = 2.4 Hz, OCH_2), 3.63 (8H, q, J = 7.2 Hz, N-CH_2CH$_3$), 2.45 (1H, t, J = 2.4 Hz, ≡C*H*), 1.33 (12H, t, J = 7.2 Hz, CH_3).

Synthesis of 1-(4-Hydroxyphenyl)-4-nitro-3-phenylbutan-1-one (aBOD1)

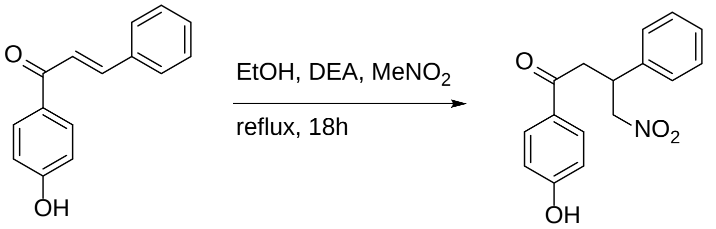

Dimethylamine (45 mmol, 3.02 g, 4.6 mL) and nitromethane (90 mmol, 5.5 g, 4.8 mL) were added to a solution of 4'-hydroxychalcone (9 mmol, 2.02 g) in 15 mL ethanol. The mixture was stirred under reflux for 18 h. The solution was cooled and acidified with 0.1 M HCl. The product was extracted with ethyl acetate and washed with 0.1 M HCl. The organic layer was dried and evaporated under vacuum. The product was purified by column chromatography (DCM:methanol 20:1, R_f = 0.42) to get the

product as colourless solid. Yield: 92%. ^{1}H-NMR ($CDCl_3$, 500 MHz, δ in ppm): 7.86 (2H, m, H_{Ar}), 7.33 (2H, m, H_{Ar}), 7.27 (3H, m, H_{Ar}), 6.86 (2H, m, H_{Ar}), 5.48 (1H, s (broad), O*H*), 4.92-4-60 (2H, m, NO_2-CH_2), 4.21 (1H, m, Ph-C*H*), 3.39 (2H, m, O=C-CH_2).

Synthesis of 5-(4-Hydroxyphenyl)-3-phenyl-1H-pyrrol-2-yl]-[5-(4-hydroxyphenyl)-3-phenylpyrrol-2-ylidene]amine (aBOD2)

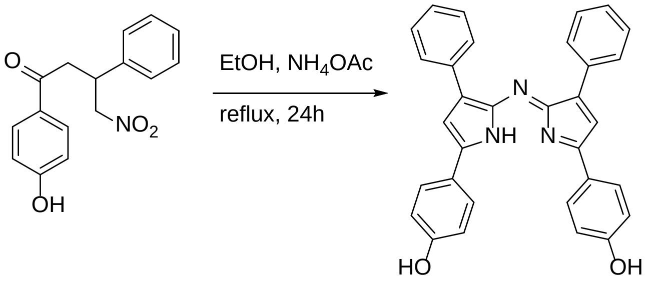

Ammonium acetate (140 mmol, 10.8 g) and aBOD1 (4.2 mmol, 1.2 g) were solved in ethanol (40 mL) and stirred under reflux for 24 h. After cooling to room temperature water (50 mL) was added and the ethanol removed under reduced pressure. The precipitate was filtered and solved in ethyl acetate. The aqueous filtrate was extracted with ethyl acetate. The combined organic layers were dried with Na_2SO_4 and the solvent removed under reduced pressure. The product was purified by column chromatography (chloroform:methanol 10:1, R_f = 0.44) to get the product as dark blue solid. Yield: 33%. ^{1}H-NMR (methanol-d4, 500 MHz, δ in ppm): 8.05 (4H, m, H_{Ar}), 7.88 (4H, m, H_{Ar}), 7.39 (4H, m, H_{Ar}), 7.32 (2H, m, H_{Ar}), 7.26 (2H, s, H_{Ar}), 6.97 (4H, m, H_{Ar}).

Synthesis of BF_2 Chelate of [5-(4-hydroxyphenyl)-3-phenyl-1H-pyrrol-2-yl]-[5-(4-hydroxyphenyl)-3-phenylpyrrol-2-ylidene]amine (aBOD3)

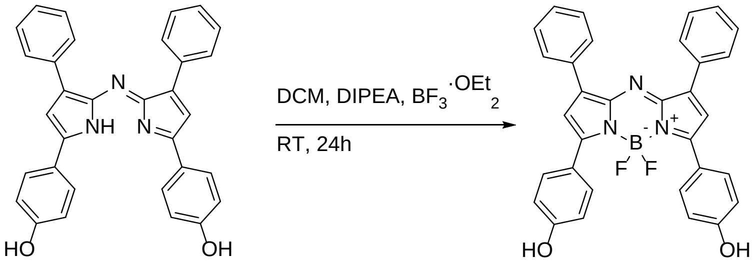

A solution of aBOD2 (0.7 mmol, 340 mg) in dry DCM (25 mL) was treated with DIPEA (7 mmol, 0.9 g, 1.2 mL) and placed in an ice bath. $BF_3 \cdot OEt_2$ (10.5 mmol, 1.49 g, 1.3 mL) was added slowly, the ice bath was removed, and the resulting reaction mixture was stirred at room temperature for 24 h. The solution was diluted with ethyl acetate, washed with water and brine, dried with Na_2SO_4 and the solvent was evaporated under vacuum. The product was purified by column chromatography (ethyl acetate:hexane 1:1, R_f = 0.36) to get the product as dark red solid. Yield: 65%. ^{1}H-NMR (methanol-d4, 500 MHz, δ in ppm): 8.15-8.07 (8H, m, H_{Ar}), 7.49-7.39 (6H, m, H_{Ar}), 7.28 (1H, s, H_{Ar}), 6.92 (4H, m, H_{Ar}).

Synthesis of BF_2 Chelate of 4-{4-phenyl-5-[3-phenyl-5-(4-prop-2-ynyloxyphenyl)-pyrrol-2-ylideneamino]-1H-pyrrol-2-yl}phenol (aBOD)

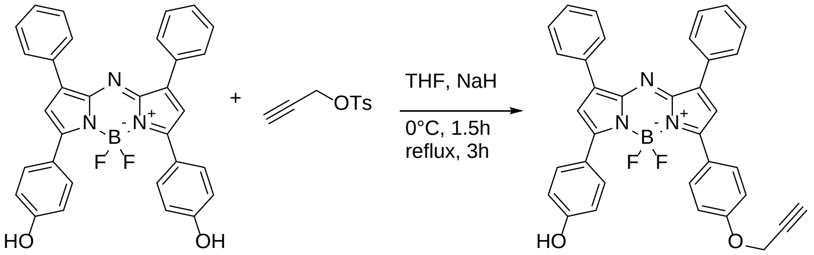

Sodium hydride (60% suspension in mineral oil, 1.125 mmol, 45 mg) and aBOD3 (0.45 mmol, 240 mg) were placed in a flask and dry THF (10 mL) was added. The stirring suspension was placed in an ice bath and propargyl tosylate (0.99 mmol, 208.15 mg, 168.95 µL) in THF (5 mL) was added. After 1.5 h the ice bath was removed, and the reaction mixture was stirred under reflux for 3 hours. The reaction was quenched with H_2O and the product extracted with ethyl acetate. The product solution was dried with Na_2SO_4 and the solvent evaporated under vacuum. The product was purified by column chromatography (ethyl acetate:hexane 2:1, R_f = 0.65) to get the product as dark red solid. Yield: 23%. ^{1}H-NMR ($CDCl_3$, 500 MHz, δ in ppm): 8.12-8.03 (8H, m, H_{Ar}), 7.50-7.40 (6H, m, H_{Ar}), 7.11 (2H, m, H_{Ar}), 7.05 (2H, s, H_{Ar}), 6.96 (2H, m, H_{Ar}), 5.26 (1H, s (broad), O*H*), 4.79 (2H, d, J = 2.4 Hz, O-CH_2), 2.58 (1H, t, J = 2.4 Hz, ≡C*H*).

Synthesis of Cyanoisopropyl dithiobenzoate (CPDB)

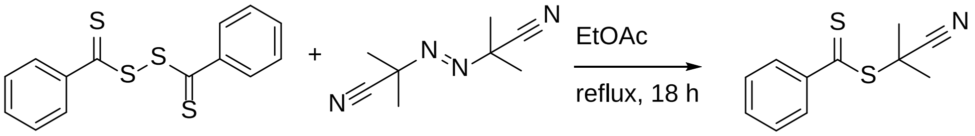

Bis(thiobenzoyl) disulfide (0.8 mmol, 245.2 mg) and AIBN (1.2 mmol, 197.1 mg) were solved in 10 mL ethyl acetate. The solution was degassed by five freeze-pump-thaw cycles and stirred under reflux for 18 h. The crude product was dried under vacuum and purified by column chromatography (hexane:ethyl acetate 10:1, R_f = 0.27) to get the product as a red oil. Yield: 56%. %. ^{1}H-NMR ($CDCl_3$, 400 MHz, δ in ppm): 7.92 (2H, m, *o*-H_{Ar}), 7.56 (1H, m, *p*-H_{Ar}), 7.39 (2H, m, *m*-H_{Ar}), 1.94 (6H, s, CH_3).

Synthesis of 3-Azido-1-propanol (APOH)

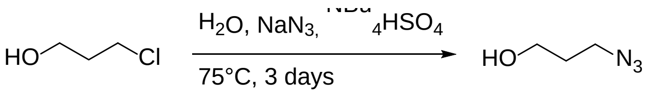

3-Chloro-1-propanol (23.5 mmol, 2.2 g, 2 mL) was added dropwise to a solution of sodium azide (48 mmol, 3.13 g) and tetrabutylammonium hydrogen sulphate (0.2 mmol, 70 mg) in 50 mL water at room temperature. The resulting solution was heated to 75°C. After stirring for three days the product was extracted with DCM. The combined organic phases were dried with Na_2SO_4 and the solvent evaporated under vacuum to get the product as a colourless liquid. Yield: 85%. %. ^{1}H-NMR ($CDCl_3$, 400 MHz, δ in ppm): 3.71 (2H, t, *J*= 6.0 Hz, HO-CH_2), 3.42 (2H, t, *J* = 6.6 Hz, CH_2-N_3), 2.21 (1H, s, O*H*), 1.80 (2H, tt, *J* = 6.6, 6.0 Hz, $CH_2CH_2CH_2$).

Synthesis of 3-Azidopropyl methacrylate (APMA)

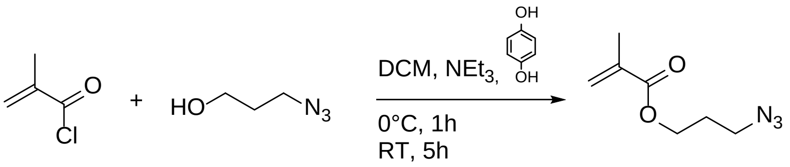

APOH (19.3 mmol, 1.95 g, 1.8 mL), triethylamine (28.7 mmol, 2.9 g, 4 mL) and hydroquinone (10 µmol, 1 mg) were solved in 25 mL of dry DCM and placed in an ice bath. Methacryloyl chloride (28.7 mmol,3 g, 2.8 mL) in 5 mL of dry DCM was added dropwise. The resulting solution was stirred at 0°C for 1 h and at room temperature for additional 5 h. The white precipitate was removed by filtration and the product solution washed with water, saturated sodium bicarbonate solution and brine. The organic phase was dried with Na_2SO_4 and the solvent was removed under vacuum. The product was purified by column chromatography (hexane:diethyl ether 2:1, R_f = 0.46) to get the product as a pale-yellow liquid. The product was stored with hydroquinone as stabilizer. Yield: 25%. ^{1}H-NMR ($CDCl_3$, 500 MHz, δ in ppm): 6.11 (1H, dq, *J* = 2.0, 1.0, =C*H*), 5.58 (1H, dq, *J* = 2.0, 1.6 Hz, =C*H*), 4.24 (2H, t, *J* = 6.2 Hz, COO-CH_2), 3.42 (2H, t, *J* = 6.7 Hz, CH_2-N_3), 1.96 (2H, tt, *J* = 6.7, 6.2 Hz, $CH_2CH_2CH_2$), 1.95 (3H, dd, *J* = 1.6, 1.0 Hz, CH_3).

Synthesis of 3-(Trimethylsilyl)propargyl methacrylate (TMSPMA)

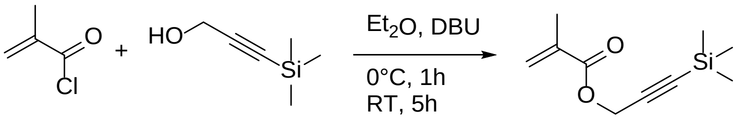

3-(Trimethylsilyl)propargyl alcohol (15.58 mmol, 2.0 g, 2.15 mL) and 1,8-diazabicyclo[5.4.0]undec-7-ene (18.7 mmol, 1.42 g, 1.4 mL) were solved in 20 mL of dry diethyl ether and cooled to 0°C. Methacryloyl chloride (18.7 mmol, 1.95 g, 1.8 mL) in 10 mL of dry diethyl ether was added dropwise. The resulting solution was stirred at 0°C for 1 h and at room temperature for additional 5 h. The yellow precipitate was removed by filtration and the product solution washed with water and brine. The organic phase was dried with Na_2SO_4 and the solvent was removed under vacuum. The product was purified by column chromatography (hexane:diethyl ether 20:1, R_f = 0.19) to get the product as colourless liquid. Yield: 30%. ^{1}H-NMR ($CDCl_3$, 400 MHz, δ in ppm): 6.17 (1H, dq, *J* = 1.0, 2.2 Hz, =C*H*), 5.61 (1H, dq, *J* = 1.6, 2.2 Hz, =C*H*), 4.76 (2H, s, OC*H*$_2$), 1.96 (3H, dd, *J* = 1.6, 1.0 Hz, C*H*$_3$), 0.18 (9H, s, Si(C*H*$_3$)$_3$).

Synthesis of Poly[(poly(ethylene glycol) methyl ether methacrylate)-co-(3-azidopropyl methacrylate)-co-(3-(trimethylsilyl)propargyl methacrylate] (Polymer I)

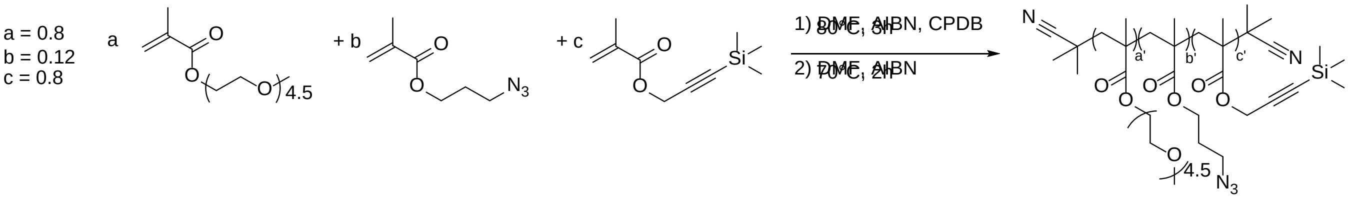

Poly(ethylene glycol) methyl ether methacrylate (Mn=300) (4.8 mmol, 1.44 g), APMA (0.72 mmol, 121.8 mg, 113.8 µL) and TMSPMA (0.48 mmol, 94.2 mg, 101.3 µL) were solved in 1 mL dry DMF in a Schlenk tube. 1 mL of a stock solution of CPDB (20 mM) and AIBN (4 mM) was added to the solution. The resulting mixture was degassed by five freeze-pump-thaw cycles and stirred at 80°C for 3 h. The product was precipitated in cold hexane:diethyl ether (2:1) as a pink polymer. To remove the RAFT-endgroup, the polymer was solved in DMF, 10 mg of AIBN were added and the solution degassed by bubbling with N_2. The solution was stirred at 70°C for 2 h. The product was again precipitated in cold hexane:diethyl ether (2:1). The resulting yellow polymer was purified by dialysis in THF. The highly viscous product was stored in DCM at 5°C to prevent auto crosslinking. Yield: 52%. GPC (THF): M_n = 36.1 kDa, M_n/M_w = 1.7. ^{1}H-NMR ($CDCl_3$, 500 MHz, δ in ppm): 4.66-4.55 (C*H*$_2$-≡-Si(CH_3)$_3$) 4.25-3.90 (COOC*H*$_2$), 3.82-3.49 (OCH$_2$C*H*$_2$O), 3.48-3.43 (N$_3$C*H*$_2$), 3.39 (OC*H*$_3$), 2.07-1.72 (C*H*$_2$), 1.12-0.77 (C*H*$_3$), 0.20 (Si(C*H*$_3$)$_3$). IR (KBr): 2178 cm^{-1} (ν_{alkyne}), 2100 cm^{-1} (ν_{N3}). Because of peak overlapping, the found values for a', b' and c' were approximated by peak integraion to be 0.83, 0.1 and 0.07 (see Figure S1c)

Synthesis of unlabelled single-chain nanoparticles (SCNP II) and labelled single chain nanoparticles (SCNP III)

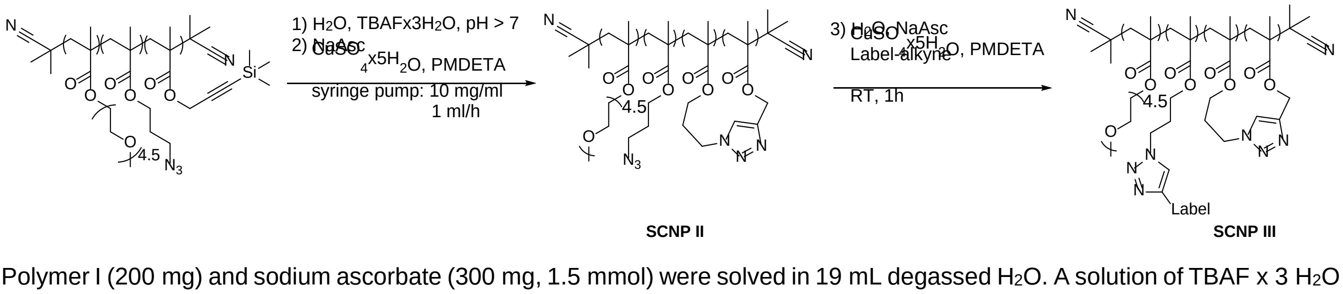

Polymer I (200 mg) and sodium ascorbate (300 mg, 1.5 mmol) were solved in 19 mL degassed H_2O. A solution of TBAF x 3 H_2O (116 µmol, 36.57 mg) and PMDETA (48 µmol, 8.3 mg, 10 µL) in 1 mL degassed H_2O was added. The resulting solution was put into a syringe pump (1 mL/h) and added to a solution of sodium ascorbate (2.76 mmol, 574.22 mg), $CuSO_4$ x 5 H_2O

(0.29 mmol, 72.37 mg) and PMDETA (0.58 mmol, 100 mg, 121 µL) in 100 mL degassed H_2O. After 20 h the solution was stirred for one additional hour. To label the resulting SCNPs a solution of sodium ascorbate (0.5 mmol, 100 mg) in 5 mL degassed H_2O was added to the reaction mixture. After 10 min a solution of 15-20 mg label in 5 mL THF was added and the reaction mixture was stirred for 2h. The product was extracted with DCM. The DCM was evaporated under vacuum and the solid product was washed multiple times first with THF/PMDETA then with THF and dried under vacuum. Yield: 54%. ^{1}H-NMR (D_2O, 500 MHz, δ in ppm): 4.32-3.90 (COOC*H₂*), 3.82-3.38 (OCH_2CH_2O), 3.30 (OCH_3), 2.07-1.66 (CH_2), 1.14-0.58 (CH_3).

Synthesis of Poly[(poly(ethylene glycol) methyl ether methacrylate)-co-(3-azidopropyl methacrylate)] (Polymer I')

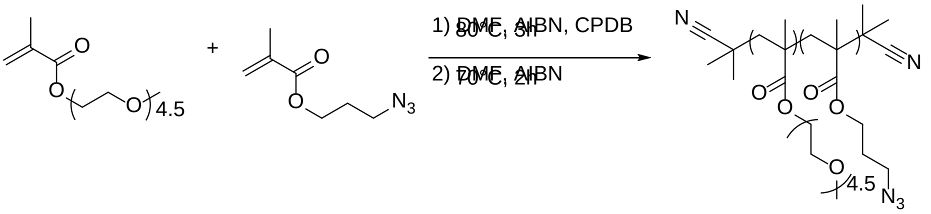

Poly(ethylene glycol) methyl ether methacrylate (Mn=300) (3.33 mmol, 1 g) and APMA (0.55 mmol, 107 mg, 100 µL) were solved in 1 mL dry DMF in a Schlenk tube. 1 mL of a stock solution of CPDB (20 mM) and AIBN (4 mM) was added to the solution. The resulting mixture was degassed by five freeze-pump-thaw cycles and stirred at 80°C for 3 h. The product was precipitated in cold hexane:diethyl ether (2:1) as a pink polymer. To remove the RAFT-endgroup, the polymer was solved in DMF, 10 mg of AIBN were added and the solution degassed by bubbling with N_2. The solution was stirred at 70°C for 2 h. The product was again precipitated in cold hexane:diethyl ether (2:1). The resulting yellow, highly viscous polymer was purified by dialysis in THF. Yield: 51%. GPC (THF): M_n = 18.2 kDa, M_n/M_w = 1.4. ^{1}H-NMR ($CDCl_3$, 500 MHz, δ in ppm): 4.25-3.90 ($COOCH_2$), 3.82-3.47 (OCH_2CH_2O), 3.48-3.40 (N_3CH_2), 3.37 (OCH_3), 2.07-1.67 (CH_2), 1.09-0.77 (CH_3).

Synthesis of Poly[(poly(ethylene glycol) methyl ether methacrylate)-co-(3-azidopropyl methacrylate)-co-(TEMPO-labelled methacrylate)] (Polymer I'a)

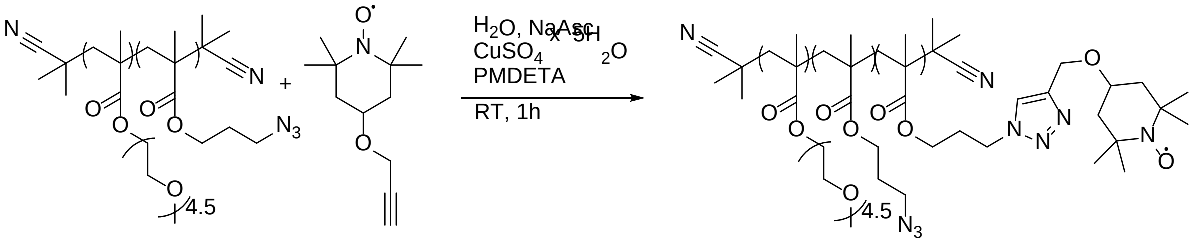

Polymer I' (50 mg, 25 µmol of N_3), TEMPO-alkyne (12.5 µmol, 2.62 mg) and sodium ascorbate (37.5 µmol, 7.4 mg) were solved in a mixture of 10 mL THF and 20 mL degassed H_2O. A solution of $CuSO_4$ x 5 H_2O (7.5 µmol, 1.9 mg) and PMDETA (15 µmol, 2.6 mg, 3.13 µL) in 5 mL degassed H_2O was added and the resulting mixture was stirred at room temperature. After 1 h the product was extracted with DCM, dried with Na_2SO_4 and the solvent removed under vacuum. The product was cleaned by dialysis in THF to get an orange polymer. Yield: 80%. ^{1}H-NMR ($CDCl_3$, 500 MHz, δ in ppm): 5.10 (triazole-C*H*), 4.25-3.90 ($COOCH_2$), 3.82-3.47 (OCH_2CH_2O), 3.48-3.40 (N_3CH_2), 3.37 (OCH_3), 2.07-1.67 (CH_2), 1.09-0.77 (CH_3).

Synthesis of Poly[(poly(ethylene glycol) methyl ether methacrylate)-co-(3-azidopropyl methacrylate)-co-(aBOD-labelled methacrylate)] (Polymer I'c)

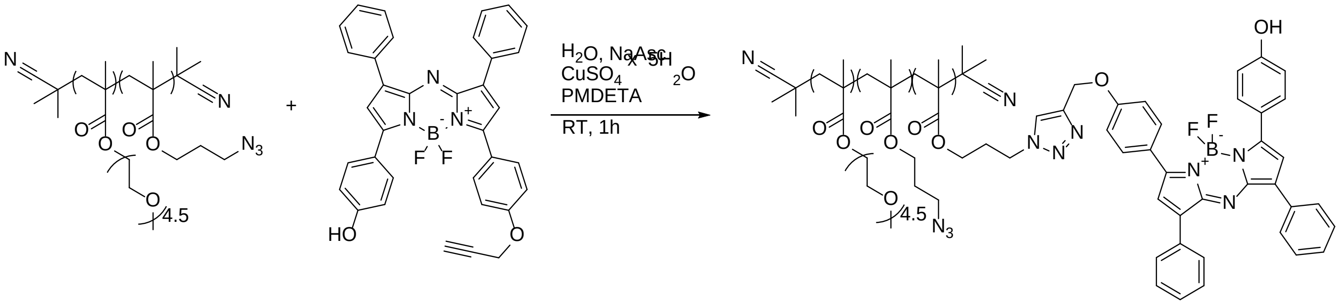

Polymer I' (50 mg, 25 µmol of N_3), aBOD (12.5 µmol, 7.09 mg) and sodium ascorbate (37.5 µmol, 7.4 mg) were solved in a mixture of 10 mL THF and 10 mL degassed H_2O. A solution of $CuSO_4$ x 5 H_2O (7.5 µmol, 1.9 mg) and PMDETA (15 µmol, 2.6 mg, 3.13 µL) in 5 mL degassed H_2O was added and the resulting mixture was stirred at room temperature. After 1 h the product was extracted with DCM, dried with Na_2SO_4 and the solvent removed under vacuum. The product was cleaned by dialysis in THF to get a green polymer. Yield: 64%. ^{1}H-NMR ($CDCl_3$, 500 MHz, δ in ppm): 8.03 (H_{Ar}), 7.41 (H_{Ar}), 7.05 (H_{Ar}), 6.95 (H_{Ar}), 5.26 (triazole-C*H*), 4.49 (triazole-CH_2-O), 4.25-3.90 (COOCH_2), 3.87-3.47 (OCH$_2$CH_2O), 3.47-3.41 (N$_3$CH_2), 3.38 (OCH_3), 2.11-1.59 (CH_2), 1.14-0.67 (CH_3).

Solving aBOD in water

aBOD (10 µmol, 5.67 mg) was solved in dry THF (2 mL) and Kolliphor EL (0.2 mL) was added. The resulting solution was ultrasonicated for 1 h. The THF was removed under vacuum and the residue was solved in 25 mL water to get a dark green solution.

Dispersing of SCNPs in water

The solid (agglomerated) SCNP were placed in a vial and water was added. The suspension was stirred for 18 h until a milky dispersion was formed. The resulting dispersion was ultrasonicated for several hours until it was clear. It is important for the temperature not to rise higher than 50°C. The SCNPs show LCST-behaviour in water and will not solubilize then.

Absorption and fluorescence measurements of aBOD

Since aBOD is a pH-responding dye, the absorption and fluorescence measurements were made in phosphate buffered water. As buffer potassium dihydrogen phosphate and potassium hydrogenphosphate were dissolved in water in a ratio that a 2 M solution of the wanted pH-value was achieved. For the measurements, 20 µL of a dye solution was mixed with 1980 µL of the aqueous buffer.

Supporting Figures

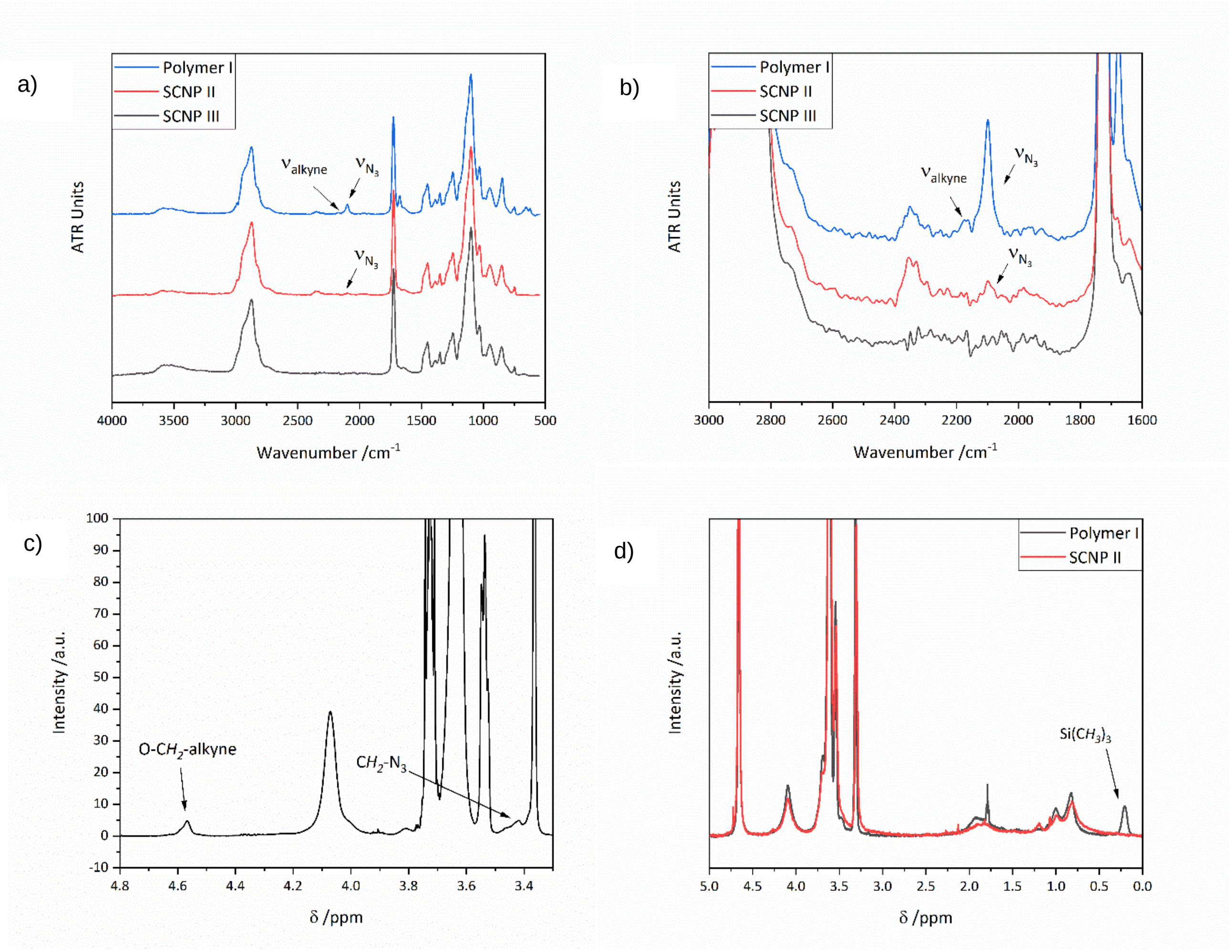

Figure S1. Proof of crosslink reaction. Comparison of ATR-IR-spectra of **Polymer I**, **SCNP II**, and **SCNP IIIb** a) complete spectra, b) bands of the reactive groups. c) ^{1}H-NMR of **Polymer I** in $CDCl_3$. d) Comparison of ^{1}H-NMR-spectra of **Polymer I** and **SCNP II** in D_2O.

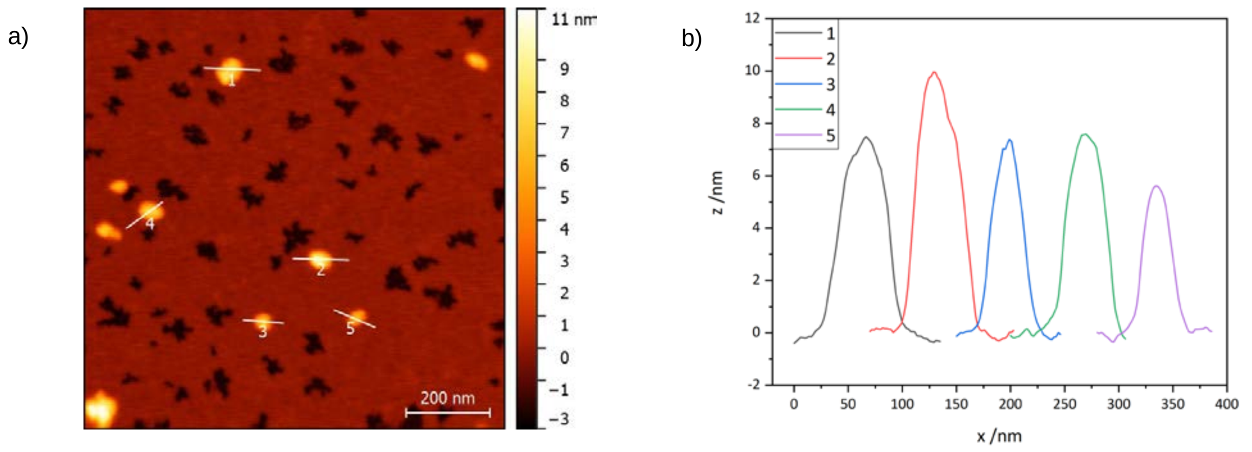

Figure S2. a) 2D AFM topography image of **SCNP IIIb** (1 x 1 µm). b) Hight profile of the highlighted particles.

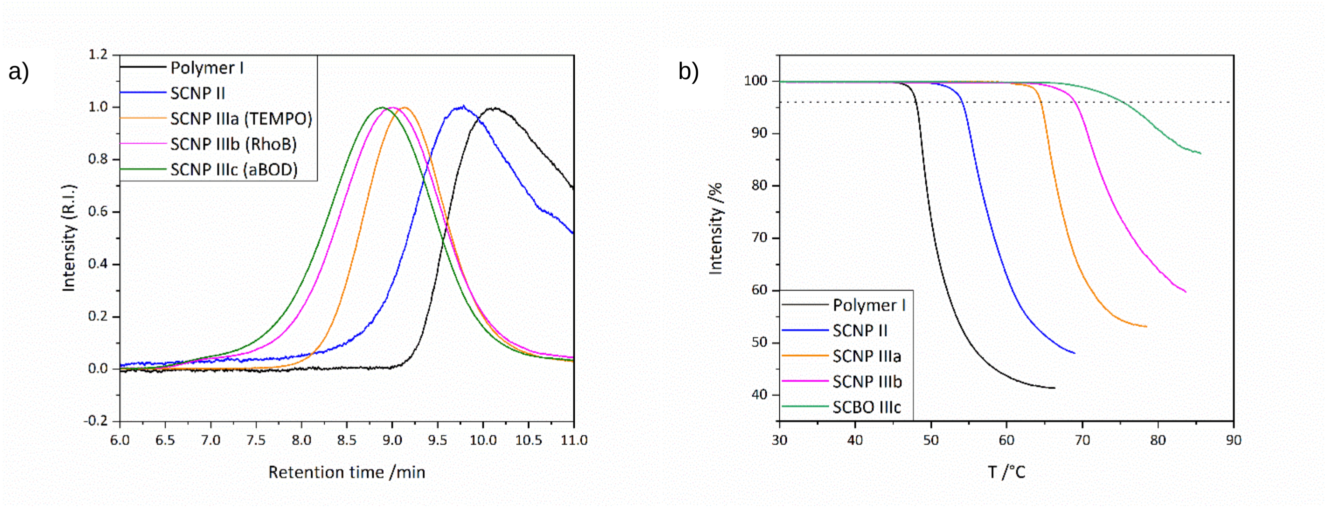

Figure S3. a) Water based SEC traces (R.I. detector) of **polymer I**, the unlabelled **SCNP II** and labelled **SCNP IIIa-c**. The peaks were normalized to the peak maxima. The complete and non-normalized chromatograms are depicted in Figure S4. b) Turbimetry measurements of **polymer I**, the unlabelled **SCNP II** and labelled **SCNP IIIa-c** in water. The polymer and nanoparticle concentrations were 1 mg/mL. T_{cp} was set as the temperature at which the light intensity was decreased to 96%.

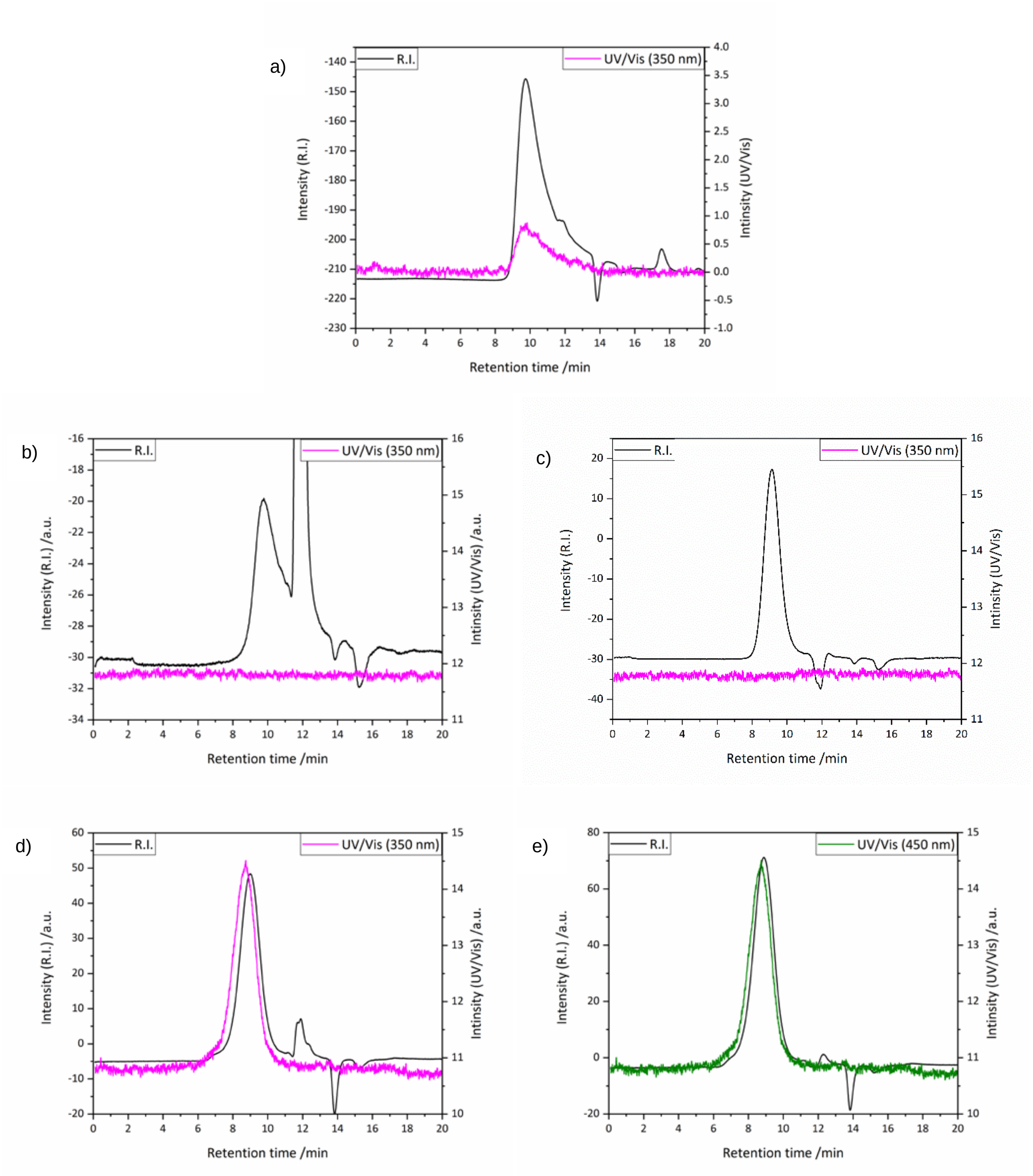

Figure S4. R.I. and UV/Vis traces of the GPC measurements in water of a) **polymer I**, b) the unlabelled **SCNP II**, c) the TEMPO labelled **SCNP IIIa**, d) the RhoB-labelled **SCNP IIIb**, and e) the aBOD-labelled **SCNP IIIc**. The system peaks start at 11.2 min.

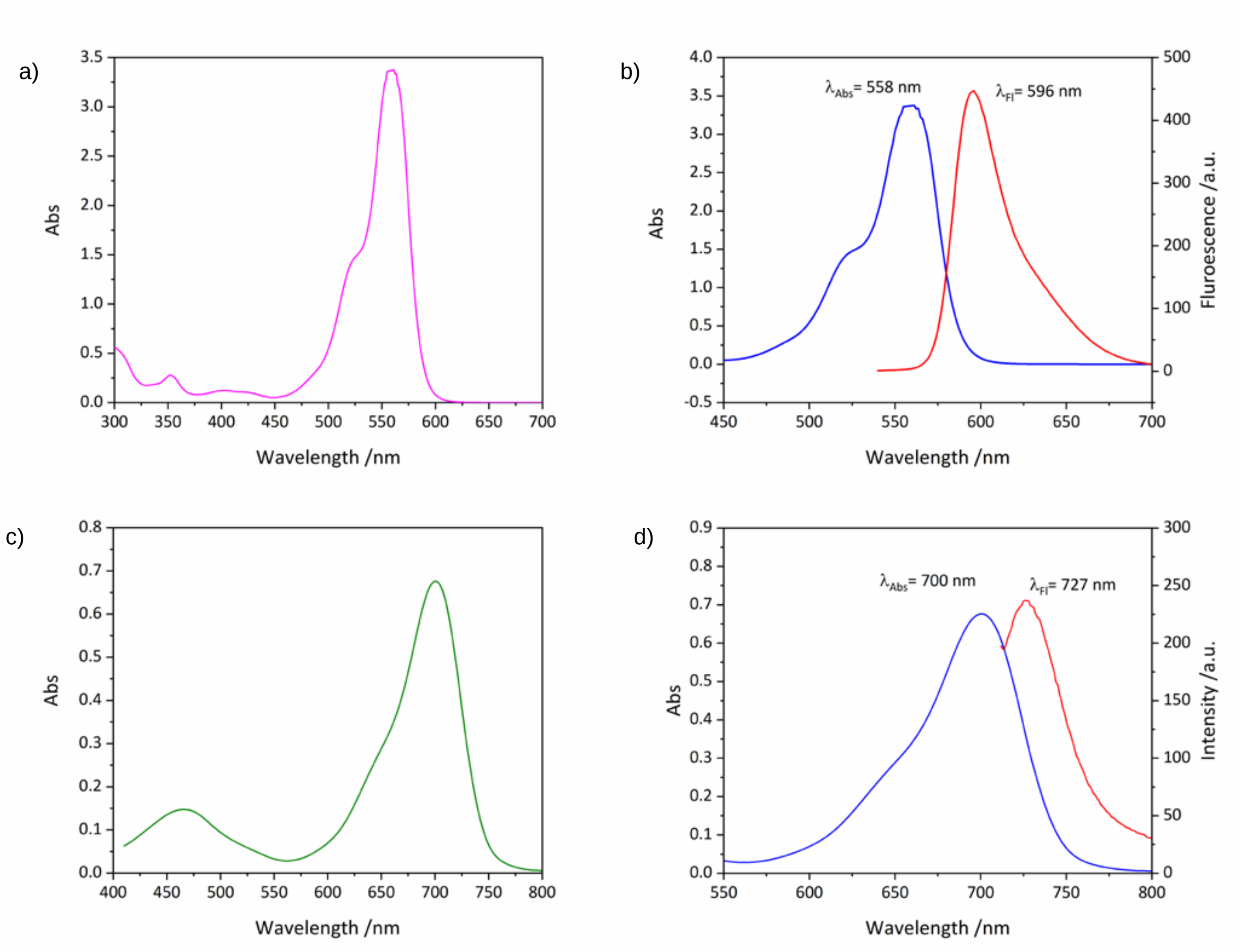

Figure S5. a) Absorption spectrum of RhoB-alkyne in water (c = 5·10^{-5} M). b) Fluorescence spectrum of RhoB-alkyne in water (c = 5·10^{-5} M, λ_{Ex} = 525 nm, slit = 5 nm, $V_{detector}$ = 500 V). c) Absorption spectrum of aBOD in aqueous phosphate buffer (pH = 5.8, c = 10^{-5} M). b) Fluorescence spectrum of aBOD in aqueous phosphate buffer (pH = 5.8), c = 10^{-6} M, λ_{Ex} = 680 nm, slit = 20 nm, $V_{detector}$ = 600 V).

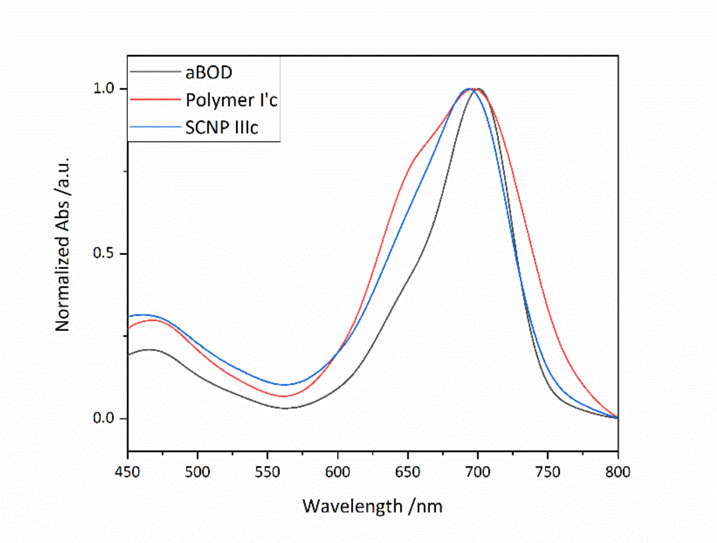

Figure S6. Normalized absorption spectra of aBOD, **polymer I'c** and **SCNP IIIc** in water.

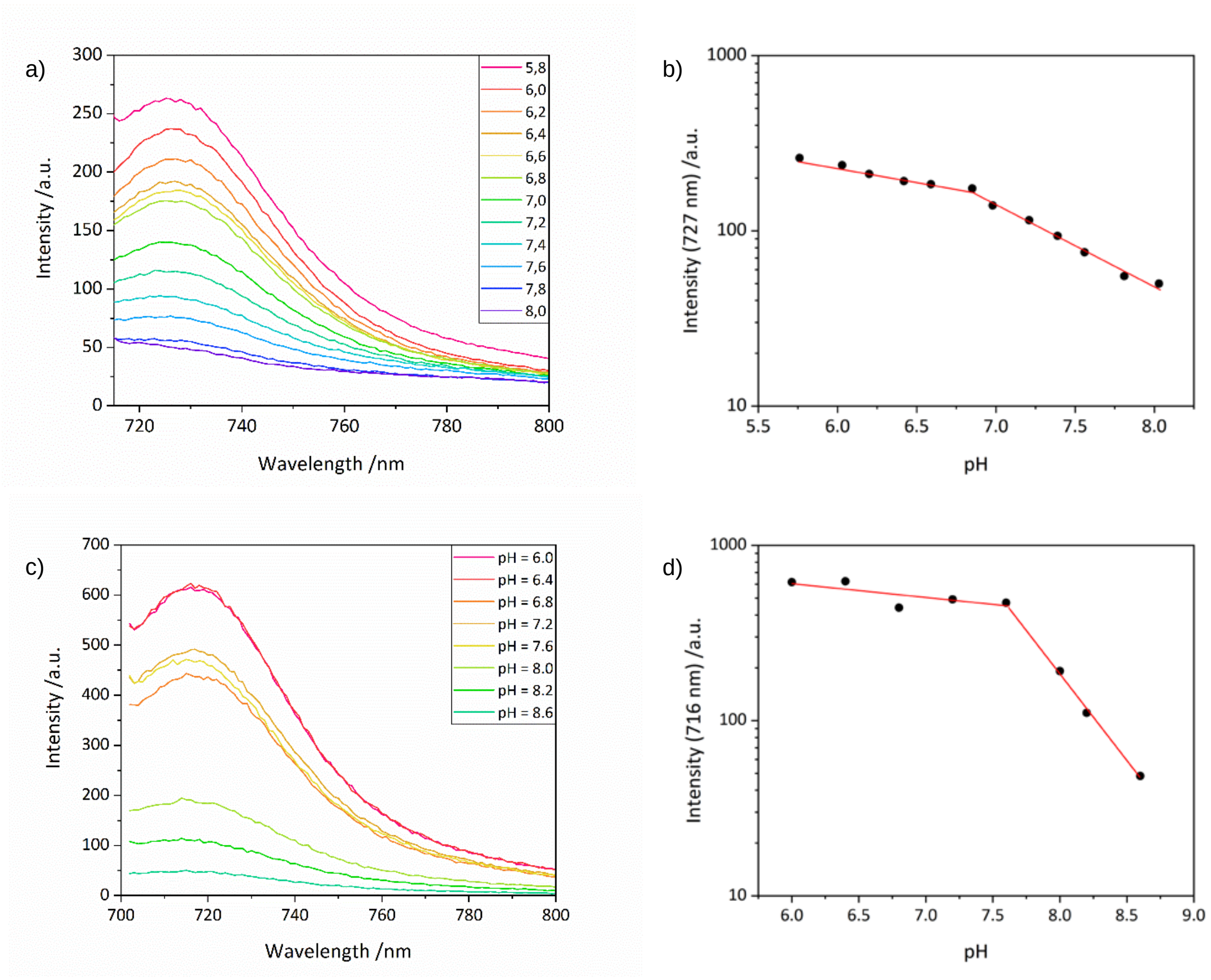

Figure S7. a) Fluorescence spectra of aBOD in aqueous phosphate buffers at different pH values (c = 10^{-6} M, λ_{Ex} = 680 nm, slit = 20 nm, $V_{detector}$ = 600 V). b) pH-plot of the fluorescence maxima of aBOD in aqueous phosphate buffers. c) Fluorescence spectra of **SCNP IIIc** in aqueous phosphate buffers at different pH values (c = 0.5 mg/mL, λ_{Ex} = 694 nm, slit = 5 nm, $V_{detector}$ = 600 V). d) pH-plot of the fluorescence maxima of **SCNP IIIc** in aqueous phosphate buffers.

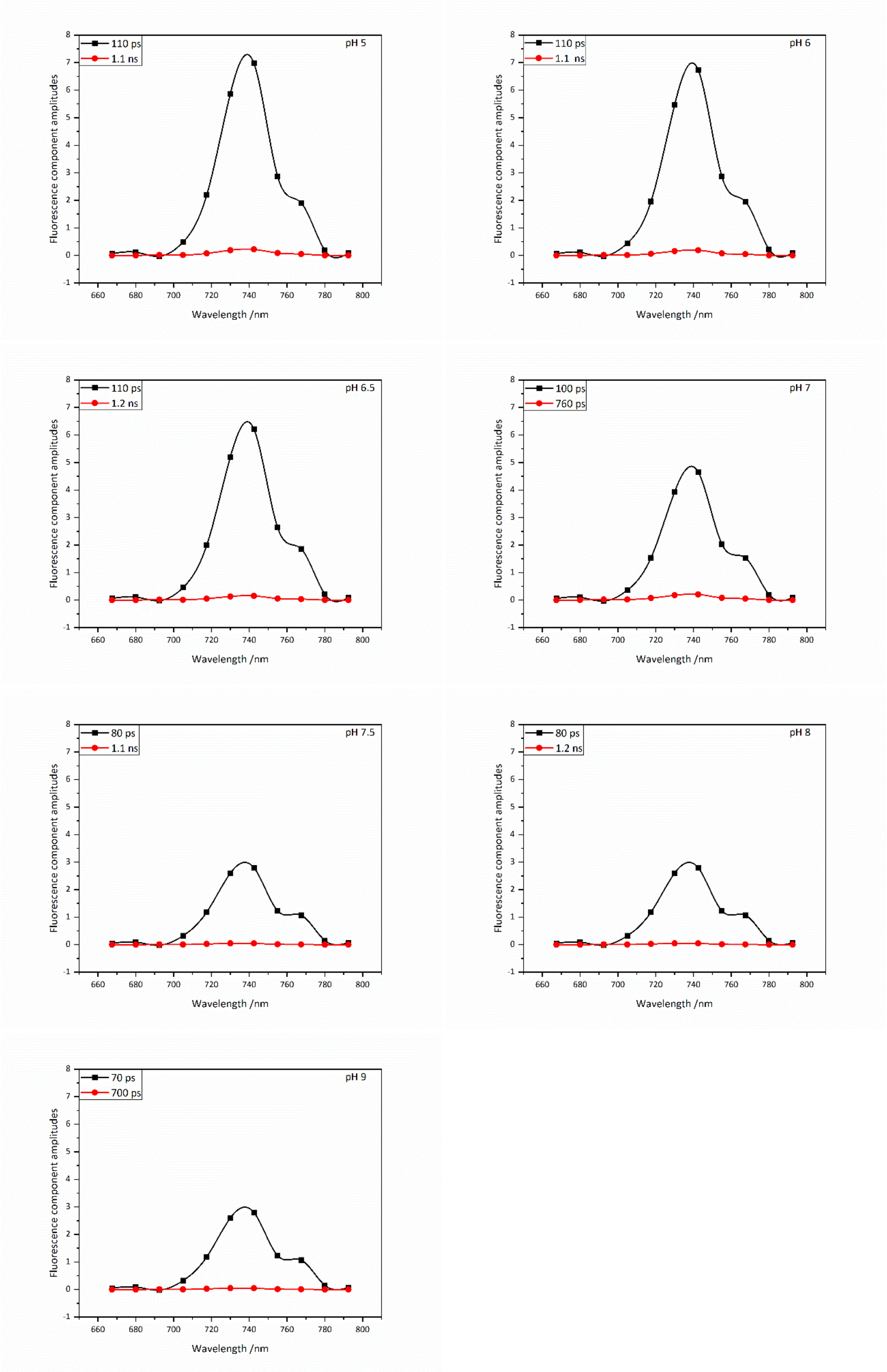

Figure S8. Decay associated spectra measured on aBOD in phosphate buffers at different pH values with 3 exponential functions.

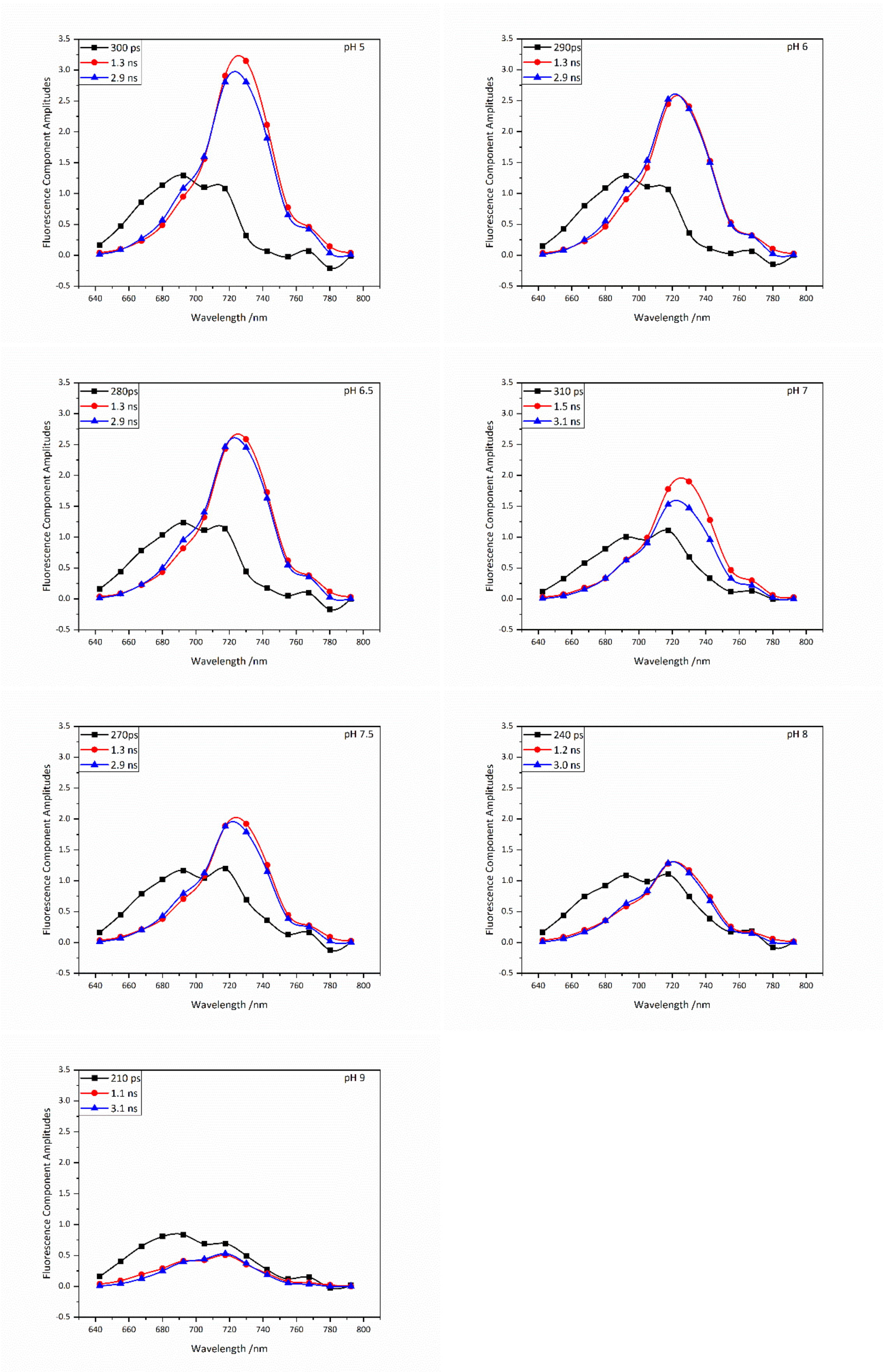

Figure S9. Decay associated spectra measured on SCNP IIIc in phosphate buffers at different pH values with 3 exponential functions.

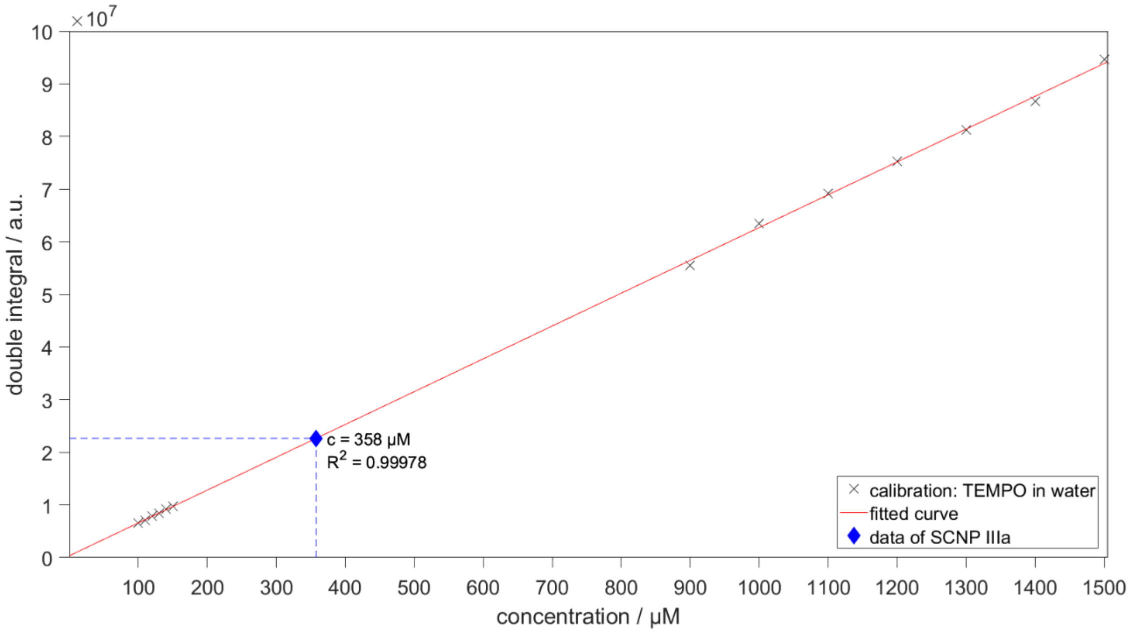

Figure S10. Calibration curve of TEMPO spin probes in water for estimation of the concentration of spin-labels in **SCNP IIIa**.

A concentration series of TEMPO spin probes in water has been used for calibration and approximate quantification of the amount of spin labels in **SCNP IIIa**. The best signal to noise ratio is given for the sample of 10 mg/ml **SCNP IIIa** (see Figure 3a) with a calculated particle concentration of 277 µM. The concentration ratio of labelled TEMPO to **SCNP IIIa** is larger than one (358 µM at a nominal particle concentration of 277 µM) so that it can be concluded that statistically 1.3 active spin labels are distributed onto the SCNP. Considering the spin-exchange frequency of ~ 6 MHz, one can safely assume that most of the spin-labeled samples that are detected bear one or two active spin labels, the former leading to the general shape of the spectra and the latter adding the moderate spin-exchange frequency due to close contact in the openly solvated or collapsed SCNP.

References

[1] F.-J. Schmitt, Z. Y. Campbell, M. Moldenhauer, T. Friedrich, *Journal of Photochemistry and Photobiology A: Chemistry* **2020**, *403*, 112838

[2] F.-J. Schmitt, Z. Y. Campbell, M. V. Bui, A. Hüls, T. Tomo, M. Chen, E. G. Maksimiv, S. I. Allakhverdiev, T. Friedrich, *Photosynth. Res.* **2019**, *139*, 185-201
